# Incidence rate of tuberculosis among HIV infected children in Ethiopia: systematic review and meta-analysis

**DOI:** 10.1186/s12887-024-04819-7

**Published:** 2024-05-24

**Authors:** Desalegn Girma, Zinie Abita, Nigusie Shifera, Mulugeta Wodaje Arage, Biruk Beletew Abate, Melsew Setegn Alie, Gossa Fetene Abebe

**Affiliations:** 1 Department of Midwifery, College of Health Science, Mizan-Tepi University, Mizan-Teferi, Ethiopia; 2 Department of Public Health, College of Health Science, Mizan-Tepi University, Mizan-Teferi, Ethiopia; 3 Department of Midwifery, School of Midwifery, College of Health Science, Woldiya University, Woldiya, Ethiopia; 4 Department of Nursing, School of Nursing, College of Health Science, Woldiya University, Woldiya, Ethiopia

**Keywords:** Children, Ethiopia, HIV, Incidence, Meta-analysis, Systemic review, Tuberculosis

## Abstract

**Background:**

Tuberculosis is one the leading causes of death from a single infectious disease, caused by the bacillus mycobacterium tuberculosis. In Ethiopia, even though several primary studies have been conducted on the incidence of tuberculosis among HIV-infected children, the pooled incidence rate of tuberculosis among HIV-infected children (aged 0–14 years) is unknown. Therefore, the main objectives of this systematic review and meta-analysis are to estimate the pooled incidence rate of tuberculosis among HIV-infected children and its predictors in Ethiopia.

**Method:**

International electronic databases such as PubMed, HINARI, Science Direct, Google Scholar, and African Journals Online were searched using different search engines.  Quality of primary studies was checked using the Joanna Briggs Institute checklist. The heterogeneity of studies was tested using I-square statistics. Publication bias was tested using a funnel plot and Egger’s test. Forest plots and tables were used to present the results. The random effect model was used to estimate the pooled incidence of tuberculosis among children living with HIV.

**Result:**

A total of 13 studies were included in this systematic review and meta-analysis. The pooled incidence of tuberculosis among HIV-infected children was 3.77 (95% CI: 2.83, 5.02) per 100-person-year observations. Advanced HIV disease (HR: 2.72, 95% CI: 1.9; 3.88), didn’t receive complete vaccination (HR: 4.40, 95% CI: 2.16; 8.82), stunting (HR: 2.34, 95% CI: 1.64, 3.33), underweight (HR: 2.30, 95% CI: 1.61; 3.22), didn’t receive Isoniazid preventive therapy (HR: 3.64, 95% CI: 2.22, 5.96), anemia (HR: 3.04, 95% CI: 2.34; 3.98), fair or poor antiretroviral therapy adherence (HR: 2.50, 95% CI: 1.84; 3.40) and didn’t receive cotrimoxazole preventive therapy (HR: 3.20, 95% CI: 2.26; 4.40) were predictors of tuberculosis coinfection among HIV infected children.

**Conclusion:**

This systematic review and meta-analysis concluded that the overall pooled incidence rate of tuberculosis among HIV-infected children was high in Ethiopia as compared to the END TB strategy targets. Therefore, emphasis has to be given to drug adherence (ART and Isoniazid) and nutritional counseling. Moreover, early diagnosis and treatment of malnutrition and anemia are critical to reduce the risk of TB coinfection.

**Registration:**

Registered in PROSPERO with ID: CRD42023474956.

**Supplementary Information:**

The online version contains supplementary material available at 10.1186/s12887-024-04819-7.

## Background

Tuberculosis (TB) is one the leading causes of death from a single infectious, caused by the bacillus mycobacterium tuberculosis [1, 2]. Children living with human immunodeficiency virus (HIV) are at increased risk of acquiring tuberculosis [3]. HIV-infected persons are sixteen times more likely to be co-infected by TB disease as compared to HIV-negative persons [2].

Globally, an estimated 1.3 million children (aged 0–14 years) fell ill with TB in 2022, which covers about 12% of the total TB cases of 10.6 million [2]. HIV and TB confection is lethal, globally, around 214,000 children died from TB disease in 2022; of this, about 31,000 of the deaths were from HIV and TB confected children [4]. The burden of TB infection differs across regions. According to the Global Tuberculosis 2023 report, a higher burden of TB was reported from African and Southeast Asia regions, contributing to about 81% of global TB deaths in 2022 [4]. Africa is home to 17 countries among 30 high TB burden countries [5]. This region alone contributes to 23% of new cases and 31% of TB-related deaths [6–8]. Moreover, about one-third (322,000) of the global TB cases of children (aged 0–14 years) were contributed from the African region; with two-thirds being unreported or undiagnosed [6, 9]. In Ethiopia, TB remains a major public health concern. Of the top 30 high TB-burden countries, Ethiopia ranked twelfth [10]. In 2022, about 151,000 people fell ill with TB in Ethiopia and over 19,000 deaths occur each year due to TB [11].

To reduce the burden of TB, the World Health Organization (WHO) adopted “End TB strategies” in 2014, setting a target to reduce the incidence rate and death of TB by 90% ( less than 10 TB cases per 100, 000 population) and 95%, respectively by 2035 compared with 2015 [12]. To achieve this target, an additional intermediate milestone was endorsed for the year 2025, set to reduce the incidence and death of TB by 50% and 75% by 2025, respectively [10, 12]. Despite multiple efforts made, globally, the incidence rate of TB (new cases per 100,000 population per year) has increased by 3.9% between 2020 and 2022 from 128 in 2020 to 133 in 2022 [4]. Moreover, only 19% and 8.7% reductions in TB deaths and TB incidence were achieved between the years 2015 to 2022, respectively [4], which is far from the WHO End TB Strategy milestone of a 50% reduction in new incidence of TB by 2025 and a 75% reduction in number of deaths by 2025 [12]. The burden of TB was more catastrophic in Sub-Saharan African countries where the incidence rate among children and adolescents living with HIV was 2,017 cases per 100,000 patient years [13]. A large multicenter cohort study conducted in South Africa found an incidence rate of 4.0 TB cases per 100 person-year among HIV-positive children receiving ART [14]. In Tanzania, the incidence rate of TB among children living with HIV ranged from 1.67 to 5.2 per 100 person years [15, 16].

In Ethiopia, studies revealed that the incidence of TB among HIV-infected children varies across regions with the highest rate (9.6 per 100 person-years) in the Benshangul Gumz region [17] to the lowest rate (2 per 100 person-years)in the Amhara region [18]. Similarly, other previous primary studies have also reported inconsistent findings regarding the incidence of TB among HIV-positive children [17–26]. With this discrepancy, the pooled incidence rate of TB for children (aged 0–14 years) living with HIV is not estimated. In Ethiopia, though there is a systematic review and meta-analysis study on the incidence of TB [27], it was conducted for all people living with HIV (including for HIV positive Adults) and yet, not segregated by age. Moreover, among eleven primary studies incorporated in the previous meta-analysis [27], only four of the primary studies were conducted among HIV-positive children and used in subgroup analysis to estimate the pooled incidence of TB among HIV-positive children, which can affect the pooled estimate of TB incidence. Furthermore, since the previous meta-analysis was conducted among all people living with HIV, the predictors were not reported in age-specific manner and predictors such as ART treatment adherence and nutritional status of children have not been investigated. Thus, an age-segregated study is crucial to identify age-specific gaps and predictors of TB among HIV-positive children. Therefore, the main objectives of this systematic review and meta-analysis are to estimate the pooled incidence rate of TB coinfection among HIV-infected children and identify its predictors in Ethiopia. Thereby to develop a comprehensive strategic plan for the identified factors at the national level.

## Methods

### Search strategy

The Preferred Reporting Items for Systematic Review and Meta-Analysis Statement (PRISMA-2020) guideline was used to report the results [28].International electronic databases such as PubMed, HINARI, Science Direct, Google Scholar, and African Journals Online were searched to obtain relevant studies. Searching was done from September 29, 2023 to back 10 years to provide up-to-date pooled estimates of TB incidences among HIV-positive children. The following terms and phrases such as “Incidence ”, “Tuberculosis”, “opportunistic infection”, “HIV infection”, “ART”, “predictors”, “associated factors”, “risk factors”, “determinants”, “pediatrics”, “children” “under-five children “and “Ethiopia” were used to search studies. The Boolean search operators such as “AND” and “OR” were used separately and in combination during database searching (Additional Table 1).

### Eligibility criteria

#### Inclusion criteria

Studies conducted in Ethiopia, studies that report the incidence rate of TB among children living with HIV, studies that report the number of new TB cases among children living with HIV, studies that report the child person-years, studies published in English languages and studies available at the electronic source in the last 10 years to September 29, 2023 were included in the study.

#### Exclusion criteria

Studies that report the predictors in other than hazard ratio and citations without abstract and/or full-text, anonymous reports, editorials, and qualitative studies were excluded from the analysis.

### Data extraction

After browsing the databases, all the articles were exported to Endnote21 to identify and remove duplication. The data was independently extracted using a standardized extraction form by four authors (GF, ZA, MS, and BB).  From each study, the author’s name, publication year, the event of TB (number of TB cases), study region, study design, the total person year, incidence rate per 100 person year, follow-up time, and the predictor of TB with hazard ratios were extracted.

### Quality assessment/critical appraisal

The Joanna Briggs Institute (JBI) Critical Appraisal Checklist for cohort study was used to assess the quality of the study [29]. The qualities of the primary studies were independently assessed by two authors (MW and NS). Any discrepancy between the two authors was handled by taking the mean score of the two authors. The tool has Yes, No, Unclear, and Not Applicable options: “1” is given for “Yes” and “0” is given for other options. The scores were summed and changed to percentages. Finally, 13 studies that received a quality score of > 50% were included in this meta-analysis [17–26, 30–32] (Additional Table 2).

### Outcome measurement

The first outcome of this systematic review and meta-analysis was the incidence rate of TB among HIV-infected children in Ethiopia. The incidence rate of TB was calculated by dividing the number of children who develop new TB cases by the total child follow-up year and multiplying it by 100. Identifying the predictors of TB coinfection among HIV-infected children was the second outcome of this study. Accordingly, the hazard ratio of predictors with its 95% confidence intervals (CI) was extracted from the original studies to compute the pooled hazard ratio for the predictor of TB coinfection among HIV-positive children.

#### Advanced HIV disease

Children older than five years whose WHO clinical stages are III and IV. Whereas, children younger than five years living with HIV are considered as having advanced HIV disease, regardless of the clinical stages.

#### Mild WHO clinical stages

HIV-positive children whose WHO clinical stages are stages I and II [33].

#### ART adherence

**Good (> 95%)—**if missed doses is ≤ 2 doses of 30 doses or ≤ 3 doses of 60 doses; **Fair: (85– 94%)** if missing doses is between 3 and 4 of 30 doses or 4–9 of 60 doses; **poor: (< 85%)** if missed doses are > 5 doses of 30 doses or 10 and above doses of 60 doses of ART drug [33].

####  Nutritional status 

Underweight : Children with weight for age Z-score < − 2 standard deviation (SD), **Stunting**: (height for age Z-score < − 2 SD) [34].

### Statistical analysis

Data entry was done using Microsoft Excel 2013 and then imported into R software version 4.1.3 for further analysis. Meta-package was used to analyze the data. Heterogeneity was checked using the I-square test [35]. Heterogeneity was declared as low, medium, and high if the I^2^ value was 25, 50, and 75%, respectively [36]. Subgroup analysis was done using the duration of the follow-up period. To identify the possible source of heterogeneity univariate meta-regression analysis was done considering the sample size and the year of publication. Sensitivity analyses were done by omitting individual studies to detect the contribution of the included study for the final pooled incidence rate of TB. Funnel plot visual inspecting was done to identify publication bias. Finally, the Egger test was done to assess any significant publication bias. Further, the trim-and-fill analysis imputation was done to correct the bias.  The forest plot was used to preent the pooled incidence of tuberculosis with its 95% confidence interval. The random effect model was used to estimate the pooled incidence of tuberculosis among children living with HIV.

## Results

### Characteristics of included studies

**Fig. 1:**
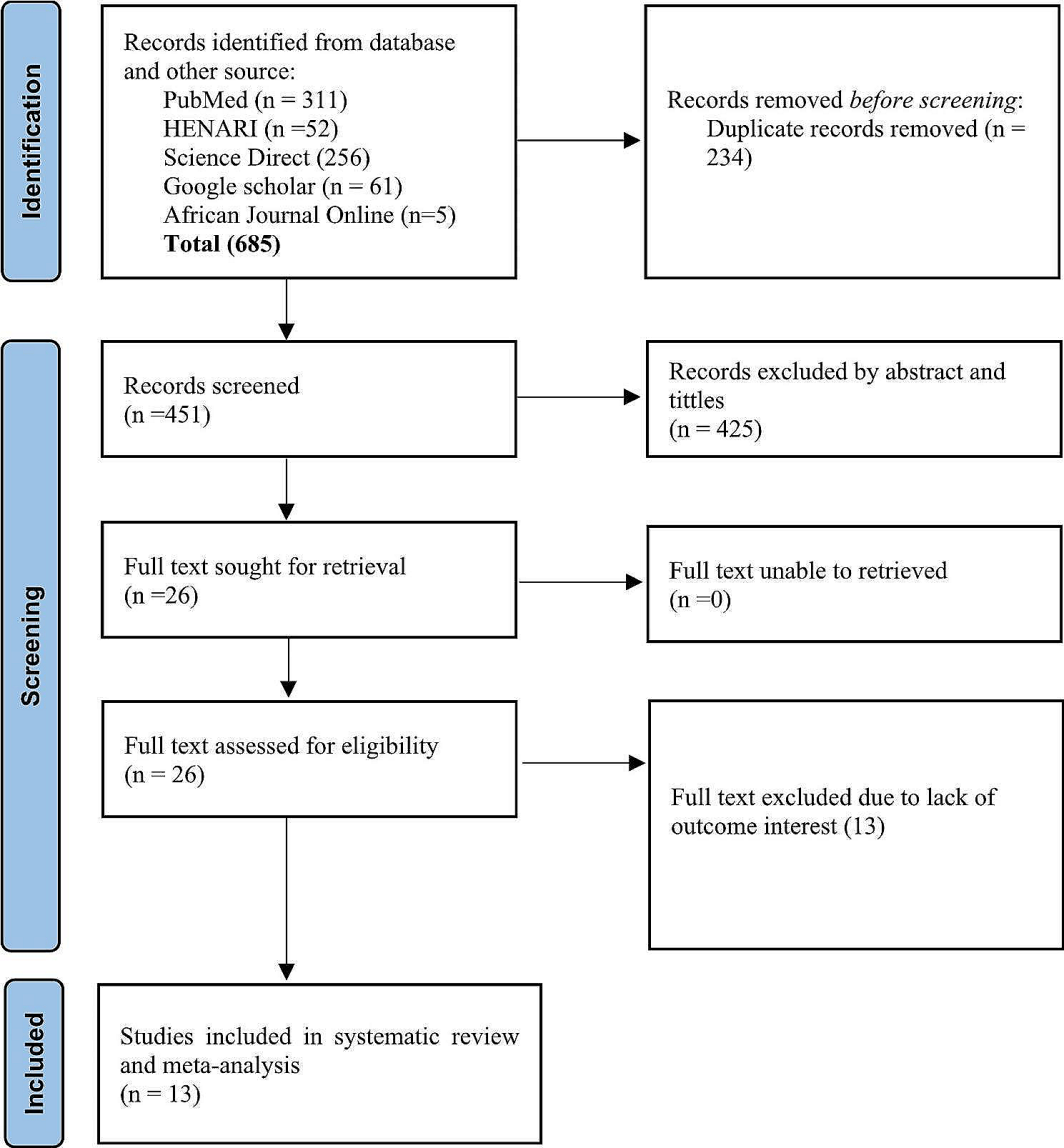
PRISMA flow chart describing screening protocols of studies for Meta-analysis

A total of 685 studies were browsed from PubMed, HINARI, Science Direct, Google Scholar, and African Journals Online. Of these, 311 studies were from PubMed, 52 studies were from HINARI, 256 articles were from Science Direct, and the rest 66 studies were searched from Google Scholar and African Journals online. From these studies, 234 articles were excluded due to duplication. From the remaining 451 articles, 425 articles were excluded as not being relevant to the study after reviewing the title and abstract. The rest 26 articles were assessed by reviewing the full text. Finally, a total of 13 studies were eligible and included in the final systematic review and meta-analysis [17–26, 30–32] (Fig. 1). All of the studies were conducted using the retrospective cohort study design. These studies were done from different parts of Ethiopia (Amhara, Oromia, SNNPR (South Nation, Nationalities and People Region), and Bnishangul Gumuz regions) (Table 1).

**Table 1:** Characteristics of studies included in the meta-analysis for the pooled incidence rate of tuberculosis among HIV infected and its predictors, Ethiopia, 2023

Primary author	Study region	Study design	Sample sizes	Tuberclosis cases	Follow-up time	Total PYO
Chanie [30]	Amhara	RFS	349	25	60	1573.5
Alemu et al. [19]	Amhara	RFS	645	79	60	1854
Wondifraw et al. [26]	Amhara	RFS	341	42	122	1802.4
Wondifraw et al. [18]	Amhara	RFS	358	60	156	2452
Kebede et al. [23]	Benishangul Gumz	RFS	721	63	60	1389.84
Tekese et al. [24]	SNNPR	RFS	371	59	82.6	1677.5
Ayalaw [20]	Amhara	RFS	271	54	72	1100.5
Beshir et al. [21]	Oromia	RFS	428	67	60	1109.6
Kebede et al. [17]	Benishangul Gumz	RFS	421	64	54	662.5
Melkamu et al. [32]	Amhara	RFS	408	42	132	1335.33
Endalamaw et al. [22]	Amhara	RFS	352	34	143	1294.7
Mekonnen et al. [31]	Amhara	RFS	452	32	92	1388.92
Tiruneh et al. [25]	SNNPR	RFS	800	233	60	2942.99

*RFS* retrospective follow-up study, *PYO* person year observation.

### The pooled incidence rate of TB among HIV‑infected children

**Fig. 2:**
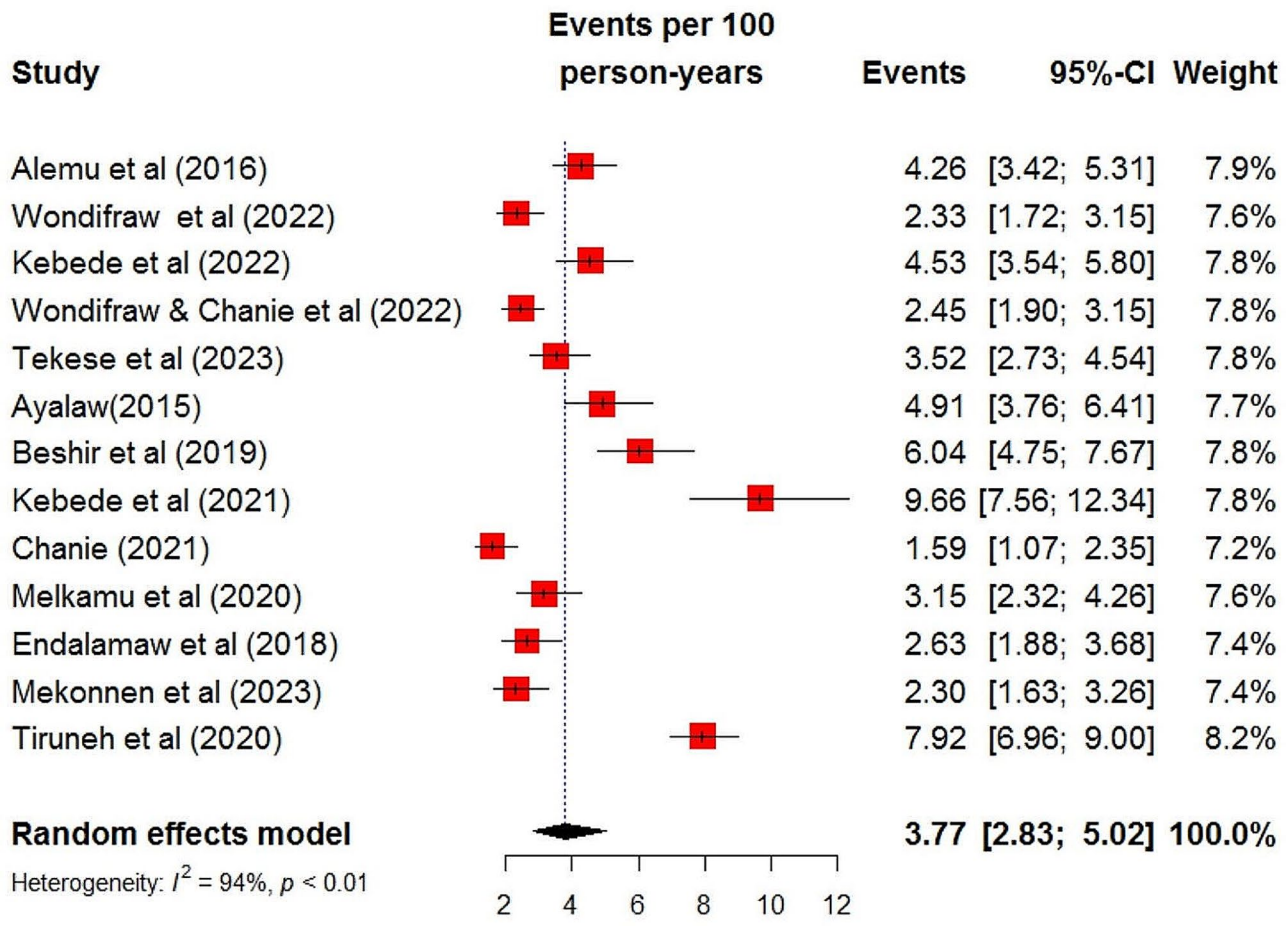
The forest plots of the incidence rate of tuberculosis among HIV-infected children in Ethiopia

**Fig. 3:**
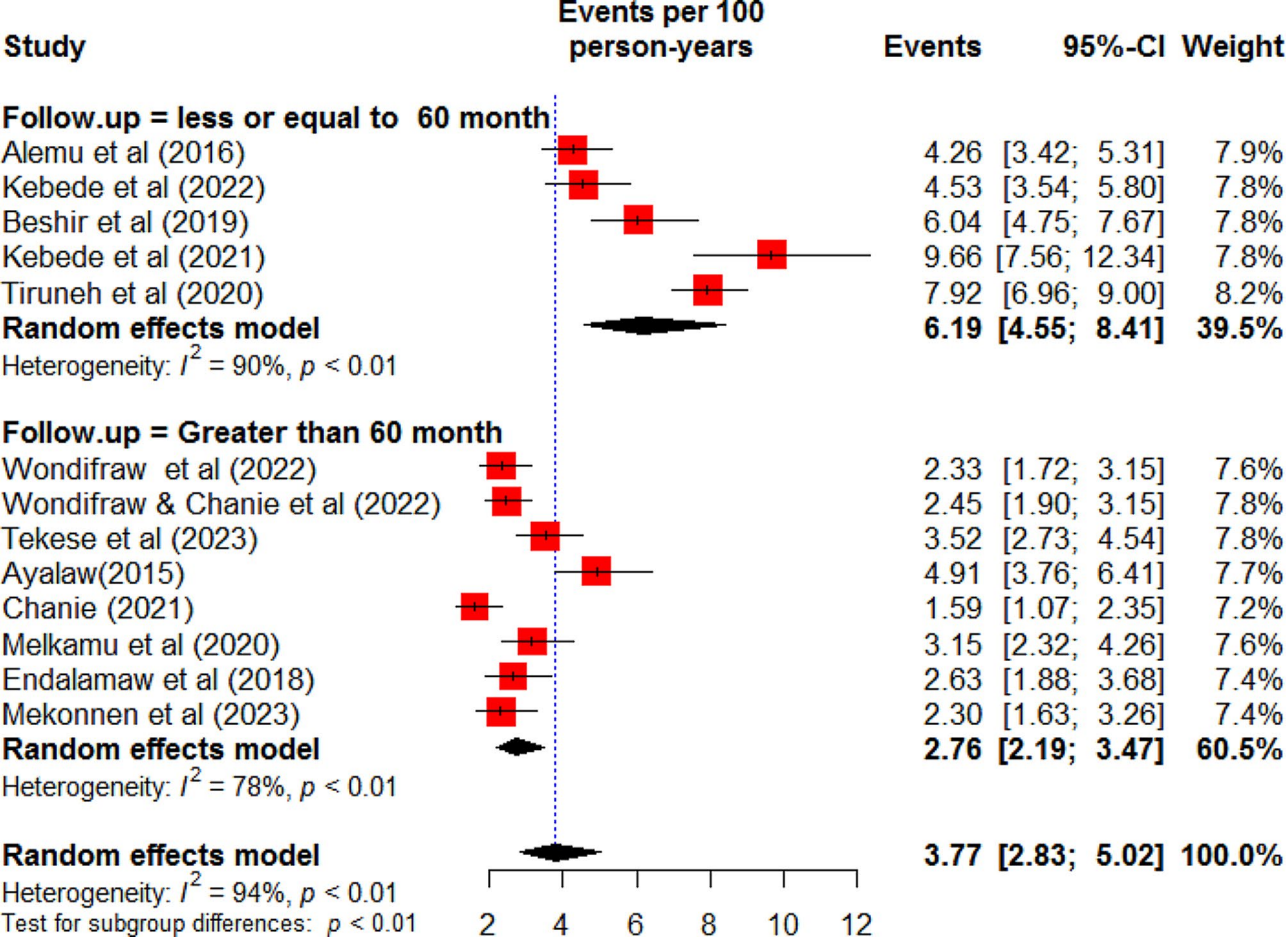
Forest plot of subgroup analysis of the incidence of tuberculosis among HIV-infected children by duration of follow up period

**Fig. 4:**
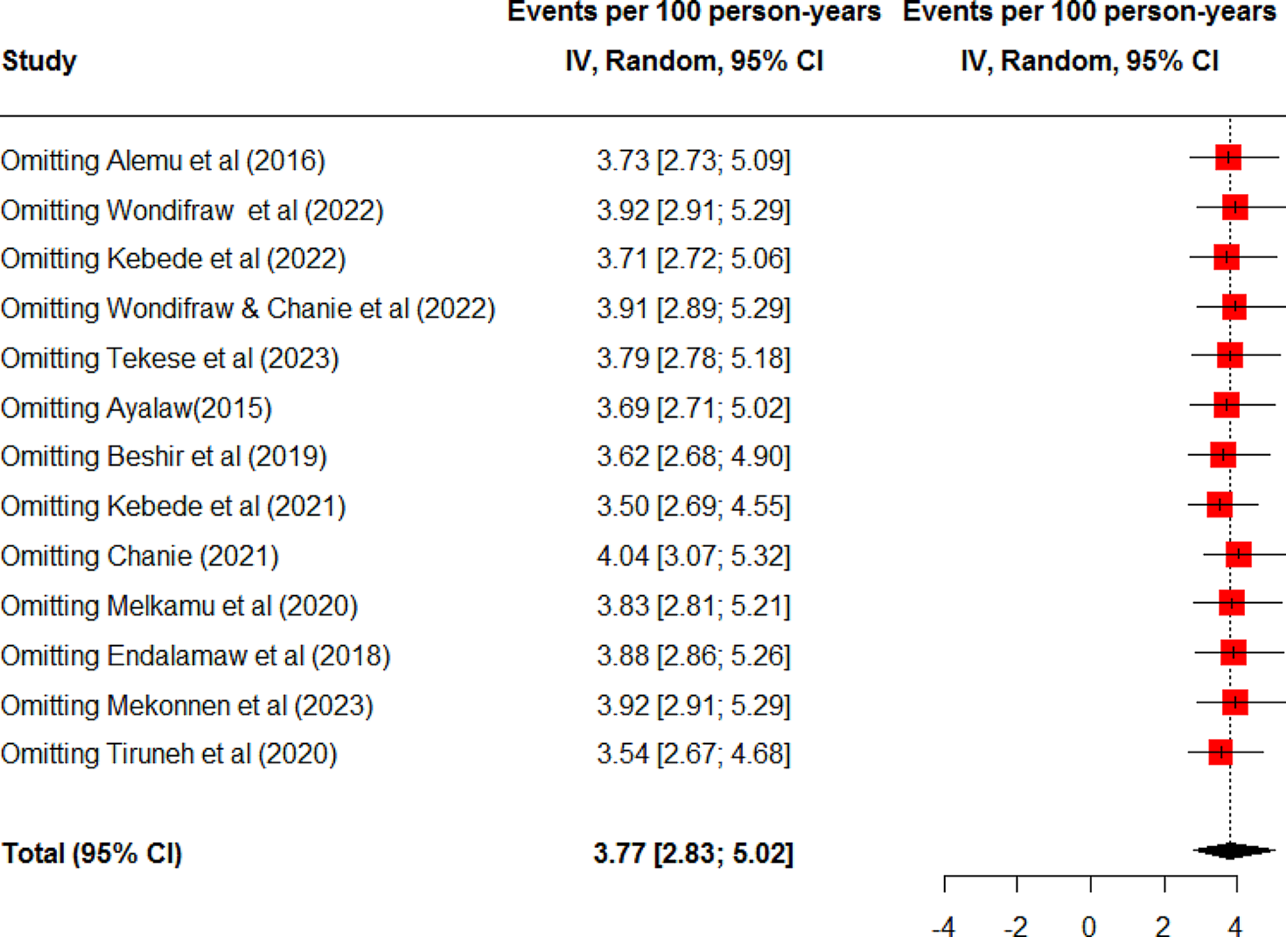
Sensitivity analysis for the incidence of tuberculosis among HIV infected children in Ethiopia

In this meta-analysis, a total of 13 studies were used to estimate the pooled incidence rate of TB among HIV-infected children in Ethiopia [17–26, 30–32]. Accordingly, the incidence rate of TB among HIV-infected children in Ethiopia was found to be 3.77 (95% CI: 2.83, 5.02) per 100-person-year observations using a random effect model. There was heterogeneity between studies included in the meta-analysis (I^2^ = 94%, P-value < 0.01 (Fig. 2). Hence, subgroup analysis was done based on the duration of follow-up time. Accordingly, the incidence rate of TB was 2.76(95% CI: 2.19, 3.47) per 100 person-years among children followed for greater than 60 months and 6.19 (95% CI: 4.55, 8.41) per 100 person-years among children followed for less or equal to 60 months (Fig. 3). Further, meta-analysis was done to identify the possible source of heterogeneity using the publication year and sample size. Accordingly, both the sample size and publication year were identified as the possible source of heterogeneity (Table 2). Sensitivity analysis was done to explore the contribution of each study for the final pooled estimate. Accordingly, except for three studies [17, 25, 30], nearly all studies have equal contributions to the pooled incidence rate of TB in Ethiopia (Fig. 4).

**Table 2:** The possible source of heterogeneity to the pooled incidence of TB-confection among HIV-positive children, Ethiopia, 2023

Variables	Coefficients	*P*-value
Publication years	–0.0547 (–0.1613, 0.0519 )	< 0.0001
Sample size	0.0010 (–0.0006, 0.0026)	< 0.0001

### Publication bias

**Fig. 5:**
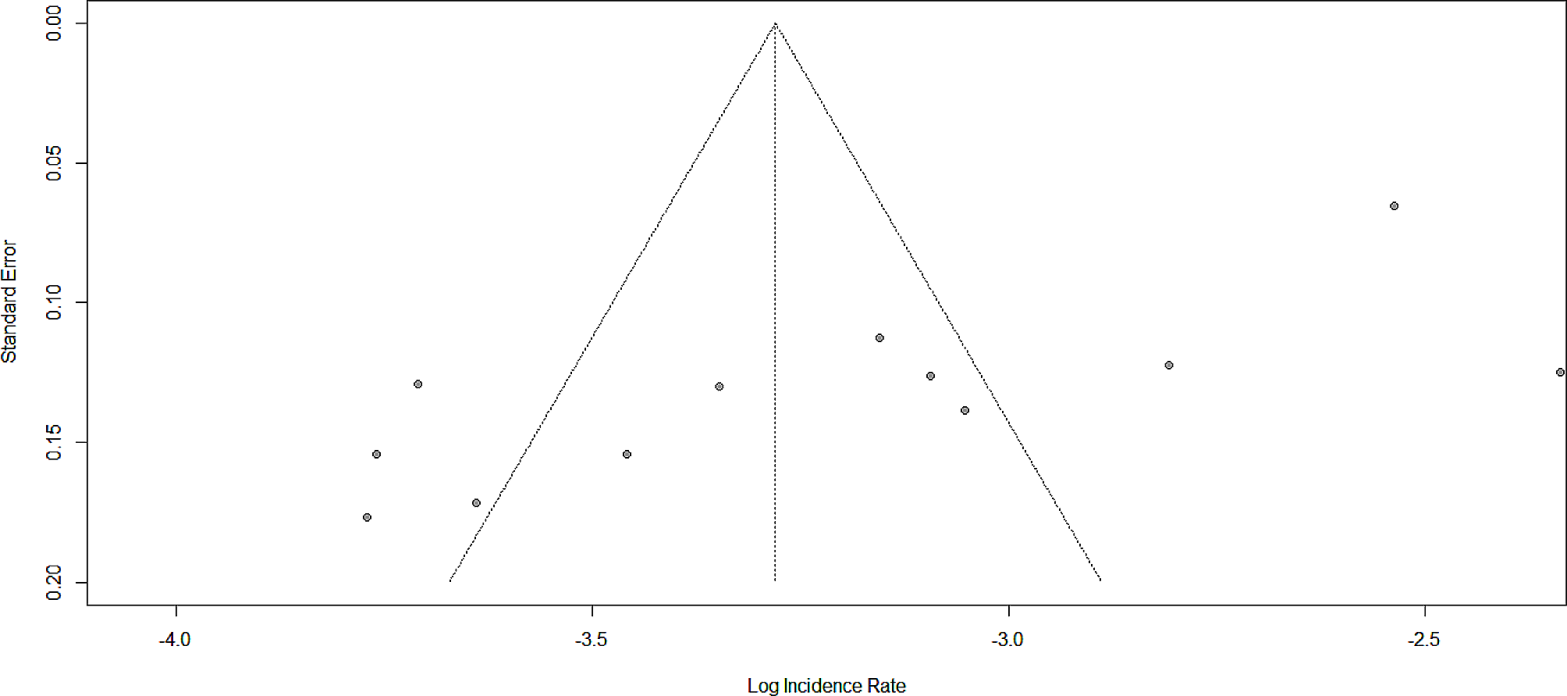
Funnel plot showing publication bias among studies used to compute the rate of tuberculosis among HIV-infected children, Ethiopia

**Fig. 6:**
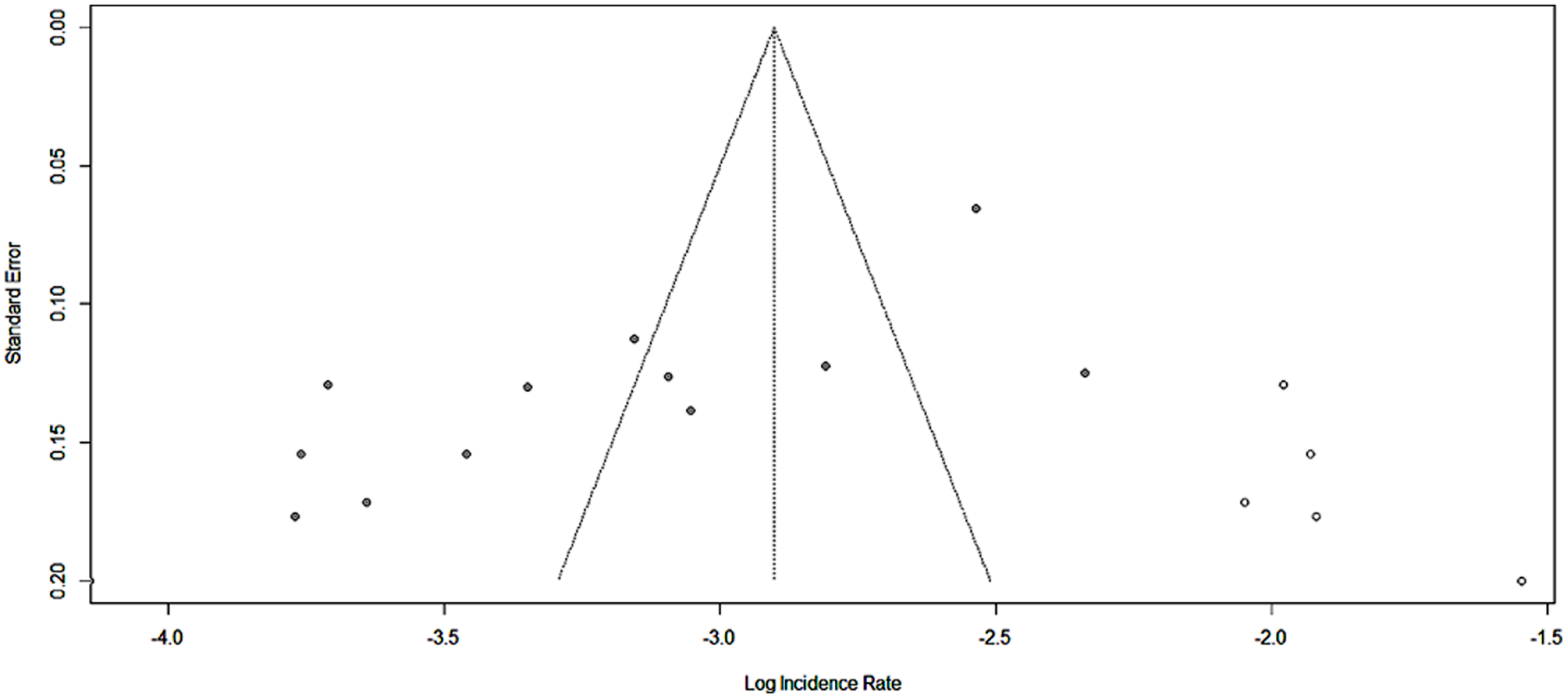
shows the trim fill analysis for the incidence rate of Tuberculosis among HIV-infected children, Ethiopia, 2023

Asymmetric distribution was detected in the funnel plot visual inspection (Fig. 5). The Egger test also shows a statistically significant publication bias with B_0_ = -2.1598, p-value = 0.03. Due to the presence of statically significant publication bias, meta-trim and fill analysis were done. Accordingly, after filling three studies, the incidence rate of TB among HIV-positive children became 5.49 (95%CI: 3.83, 7.89) per 100 child years using a random effect model (Fig. 6).

### Meta-analysis of predictors of TB among HIV-infected children in Ethiopia

A total of eight studies were used to estimate the pooled hazard ratio for predictors of TB coinfection among children living with HIV [17–24]. Accordingly, the hazard of TB coinfection among HIV-infected children was 2.72 times (HR: 2.72, 95% CI: 1.9; 3.88) higher among children with advanced HIV disease as compared to children with mild WHO clinical stages [18, 19, 22, 24]. The hazard of TB co-infection in HIV-infected children was 4.40 times (HR: 4.40, 95% CI: 2.16; 8.82) higher among children who didn’t receive complete vaccination as compared to their counterparts [17, 20, 21]. The likelihood of TB co-infection among HIV-infected children was 2.34 times (HR: 2.34, 95% CI: 1.64, 3.33) higher among children who are stunted as compared to their counterparts [17, 18, 23]. Likewise, the hazard of TB co-infection among HIV-infected children was 2.30 times (HR: 2.30, 95% CI: 1.61; 3.22) higher among children who are underweight as compared to their counterparts. The hazard of TB coinfection among HIV-infected children was 3.64 times (HR: 3.64, 95% CI: 2.22, 5.96) higher among children who didn’t receive Isoniazid preventive therapy as compared to children who received Isoniazid preventive therapy [19, 21, 23]. The likelihood of TB coinfection among HIV-infected children was 3.04 times (HR: 3.04, 95% CI: 2.34; 3.98) higher among children whose hemoglobin level is less or equal to 10 mg/dl) as compared to children whose hemoglobin level is greater than 10 mg/dl [17, 19–21, 23, 24]. . The hazard of TB coinfection in HIV-infected children was 2.5 times (HR: 2.50, 95% CI: 1.84, 3.99) higher among children whose adherence level is fair or poor as compared to children with good ART adherence [18, 22, 24]. The likelihood of TB coinfection among HIV-infected children was 3.20 times (HR: 3.20, 95% CI: 2.26; 4.40) higher among children who didn’t receive cotrimoxazole preventive therapy as compared to children who receive cotrimoxazole preventive therapy [17, 19, 21, 23] (Table 3).

**Table 3:** Meta-analysis of predictors of TB coinfection among HIV-positive children on ART, Ethiopia, 2023

Variables	Categories	Included studies	HR (95% CI)	Q-statistic	*p*-value of Q	I^2^ (%)	Tau^2^	*p*-value of estimate
WHO clinical stage	Advanced HIV disease	4	2.72 (1.9; 3.88)	1	0.61	0	0	< 0.0001
	Mild WHO clinical stages		Ref.					
ART treatment adherence	Poor/fair	3	2.50 (1.84; 3.39)	4.38	0.11	54.3	0.13	< 0.0001
	Good		Ref.					
Height for age	Stunting	3	2.34 (1.64; 3.33)	0.62	0.73	0	0	< 0.0001
	Normal		Ref.					
weight for age	Underweight	3	2.30 (1.61; 3.22)	4.19	0.12	52.3%	0.12	< 0.0001
	Normal		Ref.					
IPT	Didn’t receive	3	3.64 (2.22, 5.96)	2.94	0.23	32.0%	0.09	< 0.0001
	Received		Ref.					
CPT	Didn’t receive	4	3.20 (2.26; 4.40)	3.02	0.38	0.6	0.02	< 0.0001
	Received		Ref.					
Vaccination	Incomplete	3	4.40 (2.16; 8.82)	7.44	0.02	73.1%	0.27	< 0.0001
	Complete		Ref.					
Anemia status	Anemic	6	3.04 (2.34; 3.98)	3.26	0.66	0	0	< 0.0001
	Not anemic		Ref.					

*IPT* Isoniazid preventive therapy, *CPT* Cotrimoxazole preventive therapy, *HR* Hazard ratio

## Discussion

This systematic review and meta-analysis disclosed the pooled incidence rate of TB among HIV-infected children in Ethiopia, and further, identified its predictors. Accordingly, the incidence rate of TB among children living with HIV was 3.77 (95% CI: 2.83, 5.02) per 100-person-year. The finding is too high and requires immediate attention to achieve the End TB Strategy targets of a 90% reduction in TB incidence rate (less than 10 TB cases per 100, 000 population) by 3035 [12]. The possible elucidation for the high incidence of TB might be due to economic constraints to implement the End TB WHO strategies [37].

This systematic review and meta-analysis revealed that HIV-infected children with advanced HIV disease have a higher hazard of TB coinfection than children with mild WHO clinical stages. The finding is supported by previous studies conducted elsewhere [16, 27, 38]. The possible justification might be children with advanced HIV disease may have compromised body immunity [39]. This may trigger the progression of latent TB to disease stages.

The likelihood of TB co-infection in HIV-infected children was higher among children who are malnourished as compared to children with normal nutritional status. The finding is consistent with studies conducted elsewhere [16, 40, 41]. This is the fact that micro and macronutrients are needed to boost our immunity system [42]. Thus, being malnourished is a golden opportunity for viral replication which further compromises body immunity [43]. Finally, this can increase the incidence of TB-HIV coinfection.

The hazard of TB coinfection in HIV-infected children was higher among children whose hemoglobin level is less or equal to 10 mg/dl as compared to children whose hemoglobin level is greater than 10 mg/dl. The finding is supported by previous studies conducted elsewhere [16, 27, 38]. This could be the fact that anemia can impair the immune response and the bactericidal activity of leucocytes makes them vulnerable to infections, including tuberculosis [42, 44, 45].

This systematic review and meta-analysis revealed that HIV-infected children who didn’t receive Isoniazid preventive therapy have a higher hazard of acquiring TB coinfection than children who receive Isoniazid preventive therapy [38, 46–50]. This could be the fact that Isoniazid preventive therapy will halt the progression of latent TB from the active form of TB disease [51].

The hazard of TB coinfection in HIV-infected children was higher among children who didn’t receive cotrimoxazole preventive therapy as compared to children who received cotrimoxazole preventive therapy. The finding is consistent with previous studies conducted elsewhere [27, 38, 40, 41, 52]. This is the fact that cotrimoxazoles block the biosynthesis of nucleic acid and protein crucial to many opportunistic infections that exacerbate immunosuppression and progression of the disease [53].

In this systematic review and meta-analysis, the likelihood of TB coinfection in HIV-infected children was higher among children whose adherence level is fair or poor than children with good Antiretroviral Therapy (ART) adherence. The finding is synonymous with a previous study conducted in South Africa [54]. This is the fact that ART can halt viral replication and restore immune function and it prevents opportunistic infection, including tuberculosis [55, 56]. Such that, fair or poor ART adherence can create a golden opportunity for viral replication [57]. This can increase the risk of TB coinfection.

Lastly, this systematic review and meta-analysis revealed that HIV-infected children who didn’t receive complete vaccination have a higher hazard of TB coinfection than children who received complete vaccination. The finding is supported by a previous study conducted in Tanzania [58]. This could be that a vaccine is given to produce antibodies that defend against infectious diseases.

The clinical and public health implications of this systematic review and meta-analysis are to take prompt intervention against the identified factors and in turn to reduce the burden of TB coinfection among HIV-infected children, and finally, to reduce HIV-related child mortality. Therefore, researchers, program implementers, and policymakers should consider the aforementioned predictors in health care provision.

## Limitations

This systematic review and meta-analysis have the following limitations: In this analysis, articles published only in English were included. Only four regions were included in the analysis, such that other regions may not be represented in the study. Some predictors of TB reported only in one primary article and/or classified differently from the included articles were excluded from the analysis.

## Conclusion

This systematic review and meta-analysis concluded that the overall pooled incidence rate of tuberculosis among HIV-infected children was high in Ethiopia as compared to the END TB strategy targets. Advanced WHO clinical staging, didn’t receive complete vaccination, stunting, underweight, didn’t receive Isoniazid preventive therapy, anemia, fair or poor antiretroviral therapy adherence, and didn’t receive cotrimoxazole preventive therapy were predictors of tuberculosis coinfection among HIV infected children. Therefore, emphasis has to be given to drug adherence (ART and Isoniazid) and nutritional counseling. Moreover, early diagnosis and treatment of malnutrition and anemia are critical to reduce the risk of TB coinfection.

### Electronic supplementary material

Below is the link to the electronic supplementary material.

Supplementary Material 1

Supplementary Material 2

## Data Availability

The data is available at the corresponding author and may be provided upon request.

## Abbreviations

ART: Antiretroviral treatment
HIV: Human immunodeficiency virus
BCG: Bacillus Chalmette–Guerin
PYO: Person-year observation
TB: Tuberculosis
WHO: World Health Organization
SNNPR: South Nation Nationalities and People Region
JBI: The Joanna Briggs Institute Critical Appraisal Checklist
PRISMA: Preferred Reporting Items for Systematic Review and Meta-Analysis Statement
HR: Hazard ratio
CI: Confidence interval
