# Supplementary material for: Incidence rate of tuberculosis among HIV infected children in Ethiopia: systematic review and meta-analysis
Source: BMC Pediatr. 2024 May 24;24:363. doi: 10.1186/s12887-024-04819-7 (PMC11127285; doi:10.1186/s12887-024-04819-7)
Supplement: Supplementary file 1 — Supplementary Material 1 [file 12887_2024_4819_MOESM1_ESM.docx]

| **Database** |  | **Query** | **Items found** |
| --- | --- | --- | --- |
| PubMed | **#1** | Incidence Filters: in the last 10 years | 1,742,914 |
|  | **#2** | **Tuberculosis Filters: in the last 10 years** | 82,249 |
|  | **#3** | **opportunistic infection[MeSH Terms] Filters: in the last 10 years** | 4,972 |
|  | **#4** | **(HIV infection) OR (ART[MeSH Terms]) Filters: in the last 10 years** | 129,581 |
|  | **#5** | **(((predictors) OR (risk factors)) OR (associated factors)) OR (determinants) Filters: in the last 10 years** | 5,615,017 |
|  | #6 | ((pediatrics) OR (children)) OR (under-five children) Filters: in the last 10 years | 1,416,881 |
|  | #7 | Ethiopia Filters: in the last 10 years | 31,081 |
|  | **#8** | **#1 AND #2 OR #3 AND #4 AND #5 AND #6 AND #7** | **311** |
| HINARI |  | (Incidence) AND ((Tuberculosis) OR (opportunistic infection)) AND ((HIV infection) OR (ART)) AND ((predictors) OR (risk factors) OR (associated factors) OR (determinants)) AND ((pediatrics) OR (children) OR (under-five children)) AND (Ethiopia) | **52** |
| Science Direct |  | ("Tuberculosis" OR "opportunistic infection") AND "HIV infection" AND ("risk factor" OR "associated factors" OR "determinants" OR predictors) AND "Children" AND "Ethiopia" | **256** |
| Google Scholar |  | Incidence and predictors of Tuberculosis among children with HIV infection in Ethiopia | **28** |
|  |  | Incidence and predictors of opportunistic infection among children with HIV infection in Ethiopia | **33** |
| African journal online |  | The incidence Tuberculosis in Ethiopia | **5** |
| **Total** | | | **685** |

Additional Table 1: Search terms summary. Searched from the last 10 year to 9/29/2023
