# Supplementary material for: Incidence rate of tuberculosis among HIV infected children in Ethiopia: systematic review and meta-analysis
Source: BMC Pediatr. 2024 May 24;24:363. doi: 10.1186/s12887-024-04819-7 (PMC11127285; doi:10.1186/s12887-024-04819-7)
Supplement: Supplementary file 2 — Supplementary Material 2 [file 12887_2024_4819_MOESM2_ESM.docx]

**Additional Table 2**: critical appraisal of studies included in the systematic review and meta-analysis for pooled incidence rate of tuberculosis, Ethiopia, 2023

| Include studies | Eleven JBI Critical Appraisal Checklist for cohort Studies: The tool has Yes, No, Unclear, and Not Applicable options: “1” is given for “Yes” and “0” is given for other options | | | | | | | | | | | | | | | | | | | | | | | | | |
| --- | --- | --- | --- | --- | --- | --- | --- | --- | --- | --- | --- | --- | --- | --- | --- | --- | --- | --- | --- | --- | --- | --- | --- | --- | --- | --- |
|  | Q1 | | | Q2 | | | Q3 | | Q4 | | Q5 | | | Q6 | | Q7 | | Q8 | | Q9 | | Q10 | | Q11 | | Over all |
|  | R1 | R2 | R1 | | R2 | R1 | | R2 | R1 | R2 | R1 | R2 | | R1 | R2 | R1 | R2 | R1 | R2 | R1 | R2 | R1 | R2 | R1 | R2 |  |
| Alemu et al (2016)[ | Y | Y | Y | | Y | Y | | Y | Y | Y | Y | Y | Y | | Y | Y | Y | Y | Y | U | U | Y | Y | Y | Y | 10/11 (90.9%) |
| Wondifraw et al (2022) | Y | Y | Y | | Y | Y | | Y | Y | Y | Y | Y | Y | | Y | Y | Y | Y | Y | U | N | U | N | Y | Y | 9/11 (81.82%) |
| Kebede et al (2022) | Y | Y | Y | | Y | Y | | Y | Y | Y | Y | Y | Y | | Y | Y | Y | Y | Y | U | U | Y | Y | Y | Y | 10/11 (90.9%) |
| Wondifraw & Chanie et al (2022) | Y | Y | Y | | Y | Y | | Y | N | Y | Y | Y | Y | | Y | Y | Y | Y | Y | U | U | Y | Y | Y | Y | 10/11 (90.9%) |
| Tekese et al (2023) | Y | Y | Y | | Y | Y | | Y | Y | Y | Y | Y | Y | | Y | Y | Y | Y | Y | U | U | Y | Y | Y | Y | 10/11 (90.9%) |
| Ayalaw(2015) | Y | Y | Y | | Y | Y | | Y | Y | Y | Y | Y | Y | | Y | Y | Y | Y | Y | Y | Y | Y | Y | Y | Y | 11/11 (100%) |
| Beshir et al (2019) | Y | Y | Y | | Y | Y | | Y | Y | Y | Y | Y | Y | | Y | Y | Y | Y | Y | U | U | Y | Y | Y | Y | 10/11 (90.9%) |
| Kebede et al (2021) | Y | Y | Y | | Y | Y | | Y | Y | Y | Y | Y | Y | | Y | Y | Y | Y | Y | U | N | U | N | Y | Y | 9/11 (81.82%) |
| Chanie (2021) | Y | Y | Y | | Y | Y | | Y | Y | Y | Y | Y | Y | | Y | Y | Y | Y | Y | Y | Y | Y | Y | Y | Y | 11/11 (100%) |
| Melkamu et al (2020) | Y | Y | Y | | Y | Y | | Y | N | Y | Y | Y | Y | | Y | Y | Y | Y | Y | U | U | Y | Y | Y | Y | 10/11 (90.9%) |
| Endalamaw et al (2018) | Y | Y | Y | | Y | Y | | Y | Y | Y | Y | Y | Y | | Y | Y | Y | Y | Y | U | Y | Y | Y | Y | Y | 10.5/11 (95.4%) |
| Mekonnen et al (2023) | Y | Y | Y | | Y | Y | | Y | Y | Y | Y | Y | Y | | Y | Y | Y | Y | Y | Y | U | U | Y | Y | Y | 10/11 (90.9%) |
| Tiruneh et al (2020) | Y | Y | Y | | Y | Y | | Y | N | N | Y | Y | Y | | Y | Y | Y | Y | Y | U | U | U | U | Y | Y | 8/11 (72.72%) |
